# Compounded glucagon-like peptide-1 receptor agonists for weight loss: the direct-to-consumer market in Colorado

**DOI:** 10.1080/20523211.2024.2441220

**Published:** 2024-12-24

**Authors:** Michael J. DiStefano, Mouna Dardouri, Gina D. Moore, Joseph J. Saseen, Kavita V. Nair

**Affiliations:** a Department of Clinical Pharmacy, Skaggs School of Pharmacy and Pharmaceutical Sciences, University of Colorado Anschutz Medical Campus, Aurora, CO, USA; b Department of Neurology, School of Medicine, University of Colorado Anschutz Medical Campus, Aurora, CO, USA

**Keywords:** Advertising, direct-to-consumer, compounded, GLP-1, obesity, semaglutide, tirzepatide, weight loss

## Abstract

**Background:**

High prices and other access barriers have contributed to the rise of a market for compounded glucagon-like peptide-1 receptor agonists for weight loss in the United States. This market has not been systematically studied. We conducted a pilot study to assess the prevalence, characteristics, and advertising content of direct-to-consumer providers of compounded glucagon-like peptide-1 products for weight loss in Colorado.

**Methods:**

We conducted a cross-sectional study of websites advertising compounded glucagon-like peptide-1 products for weight loss in Colorado. Websites were identified using Google searches focused on census-defined statistical areas. Searches were conducted between March 21 and April 12, 2024. Data collected from websites included physical addresses, business type, highest reported staff credential, advertised glucagon-like peptide-1 products, whether businesses referred to Food and Drug Administration approval when describing products, and whether businesses referred to products as ‘generic’.

**Results:**

We identified 93 business websites advertising compounded glucagon-like peptide-1 products for weight loss corresponding to 188 physical locations throughout Colorado. Most businesses were self-categorized as medical/health spas (33/93) or weight loss services (26/93). Advertised products included semaglutide (92/93), tirzepatide (40/93), liraglutide (2/93), and retatrutide (1/93). Advertised combination products included B vitamins (8/93), levocarnitine (1/93), mannitol (1/93), BPC-157 (1/93), and glycine (1/93). Seven websites advertised oral formulations. Additionally, 41/93 websites referred to Food and Drug Administration approval in their descriptions of compounded products and 5/93 referred to products as ‘generic’.

**Conclusion:**

This study identified several instances of unapproved glucagon-like peptide-1 products being compounded and advertised in Colorado. Additionally, 1 product was advertised as compounded with BPC-157, a substance determined by the Food and Drug Administration to be unsafe for compounding. This study also identified numerous examples of misleading claims regarding the regulatory status of compounded glucagon-like peptide-1 products. Regulatory action is needed to ensure the benefits of compounded GLP-1 products outweigh the risks.

## Background

To date, the Food and Drug Administration (FDA) has approved three glucagon-like peptide-1 (GLP-1) receptor agonists as pharmacologic treatments for chronic weight management (Chen et al., 2023). The first to be approved for this indication was liraglutide (Saxenda) in 2014, followed by semaglutide (Wegovy) in 2021 and tirzepatide (Zepbound; a GLP-1 and glucose-dependent insulinotropic polypeptide dual agonist) in 2023 (U.S. Food and Drug Administration, 2024a). All three of these medications were originally approved to treat type 2 diabetes under different brand names. In phase 3 trials, semaglutide and tirzepatide achieved between 14.9% and 20.9% reduction in body weight after one year, as well as improvement on cardiovascular risk factors (Jastreboff et al., 2022; Wilding et al., 2021), leading to this class of drugs being hailed as ‘game changers’ for chronic weight management (Kolata, 2021).

Despite their clinical efficacy, these medications present affordability challenges for potential patients, given their current list prices between $1000 and $1400 per month (Amin et al., 2023; Goldman, 2023; GoodRx, 2024). Many commercial insurers have not yet opted to cover these drugs (Accolade, 2023) or include step therapy or prior authorisation provisions that delay eligibility and access (Wingrove, 2023). State Medicaid programmes are not required to cover weight loss drugs and, as of the first quarter of 2023, only 5 state Medicaid programmes provided unrestricted coverage of liraglutide and semaglutide for this indication and the vast majority of states provided no coverage (Liu & Rome, 2024). Finally, Medicare is prohibited by law from covering these drugs if used to treat only obesity (Cubanski et al., 2024). Without insurance coverage, many patients will be unable to afford these drugs at current list prices.

Rising demand for GLP-1 drugs despite their high price also presents barriers to access. Prescriptions for semaglutide and tirzepatide increased 2000% between 2019 and 2022 (Khan & Kim, 2023). This recent rapid increase in demand (Watanabe et al., 2023), spurred in part by celebrity endorsements touting the cosmetic benefits of these drugs (Klein, 2023), led to an FDA-designated supply shortage of liraglutide, semaglutide, and tirzepatide. The FDA first added semaglutide to its shortage list in March 2022, followed by tirzepatide in December 2022, and liraglutide in July 2023 (U.S. Food and Drug Administration, 2024a).

These access barriers have contributed to the rise of a market for cheaper, compounded GLP-1 products produced by compounding pharmacies and advertised and sold by telehealth platforms, weight loss clinics, and medical spas (Blum, 2023). Traditional 503A compounding refers to when a licensed pharmacist or physician combines, mixes, or alters the ingredients of a drug to produce a custom medication that meets the needs of an individual patient (Lisi, 2021; U.S. Food and Drug Administration, 2021). For example, patients who are allergic to a component of an FDA-approved product or require a specialised dose or dosing form may turn to a compounded drug product. When a commercially available drug product is designated by the FDA as in shortage, compounders, including 503B outsourcing facilities, are also permitted to produce ‘essentially copies’ of these products (U.S. Food and Drug Administration, 2021). In this case, compounded drugs do not need to be altered to meet the needs of individual patients and can be produced in larger quantities. Moreover, 503B facilities do not require individual prescriptions for the drugs that they produce.

Only very recently have these shortages begun to be resolved. The FDA initially removed tirzepatide from its shortages list in early October 2024. However, the FDA is now reevaluating this decision in response to a lawsuit from a compounding trade group and will permit compounding of copies of tirzepatide in the meantime (U.S. Food and Drug Administration, 2024b). Additionally, the FDA announced on October 30, 2024 that it now considers all presentations of semaglutide to be available, but similar legal challenges from the compounding industry should be expected. It is also difficult to predict how restrictions on compounding might increase demand for branded GLP-1 products, especially if more insurers begin to cover these drugs for weight loss, potentially leading to shortages again in the future. Even if GLP-1 drugs are successfully removed from and remain off the FDA shortage list for any substantial period of time, compounders may be able to legally continue offering compounded GLP-1 products so long as they are modified in some way to meet an individual patient need, for example by adding a B vitamin or personalising the dosage (Palmer & Florko, 2024).

While compounded GLP-1 products may certainly benefit some patients seeking to lose weight who are unable to afford or access the branded products, questions regarding their potential safety and effectiveness exist. Compounded drugs are not reviewed or approved by the FDA as safe and effective (U.S. Food and Drug Administration, 2023a). While 503B outsourcing facilities are subject to federal Current Good Manufacturing Practice (CGMP) regulations and must register with the FDA, traditional 503A compounders are exempt from CGMP regulations and are instead overseen primarily by state boards of pharmacy, potentially resulting in a patchwork of manufacturing regulation and oversight across states (Lisi, 2021; U.S. Food and Drug Administration, 2021). The quality of compounded GLP-1 products may therefore be variable. In the past, patient infections and deaths have been linked to sterility issues in the manufacturing processes of compounding facilities (Outterson, 2012). Regarding GLP-1 products specifically, both novel and increased levels of impurities have been identified by Novo Nordisk in compounded versus originator semaglutide samples, posing some risk of immunogenicity (Hach et al., 2024). Compounded products also carry a risk of dosing errors arising from problems with drug uniformity or dissolution (Lisi, 2021; Watson et al., 2021) or by relaxed labelling requirements for compounders that do not require the provision of adequate directions for use (U.S. Food and Drug Administration, 2021). While branded GLP-1 products are provided in dose-specific auto-injector devices, compounded versions are typically dispensed in multi-dose vials. Patients using the compounded product must withdraw and inject each dose, which is subject to user error and potential confusion regarding units versus milligrams if an insulin syringe is used for administration (U.S. Food and Drug Administration, 2024c). US poison centres reported a steep increase in 2023 of accidental overdoses of semaglutide resulting in persistent vomiting that they attributed in part to the use of compounded versions of the drug (Goodman, 2023). The FDA has also issued a warning about counterfeit semaglutide products in the US drug supply (U.S. Food and Drug Administration, 2023b), which could include some products advertised as compounded semaglutide.

The nature and extent of the market for compounded GLP-1 products to achieve weight loss has not been systematically studied. Reports have indicated that some compounders are producing semaglutide salts (e.g. semaglutide sodium and semaglutide acetate) (Blum, 2023; Goodman, 2023), a practice that does not comply with the requirements for compounding outlined in the Federal Food, Drug, and Cosmetic (FD&C) Act (U.S. Food and Drug Administration, 2021, 2024d). Reports also indicate that compounded products are being combined with B vitamins or levocarnitine, additives that are claimed to bolster the drug’s impact on weight loss, but may introduce new interactions impacting safety or effectiveness (Blum, 2023). The prevalence of these salt forms and combinations in the market is unknown. Furthermore, whether and how providers advertise these compounded products to potential consumers is unknown. A previous analysis of online direct-to-consumer advertising of off-label and unapproved ketamine treatments found false statements regarding FDA approval of ketamine and ketamine’s addictive potential (Crane et al., 2023).

In this cross-sectional analysis, we assessed the prevalence, characteristics, and advertising content of direct-to-consumer providers of compounded GLP-1 products for weight loss in the state of Colorado.

## Methods

To identify websites associated with businesses advertising these products to consumers in Colorado, we conducted location-based Google searches for ‘weight loss’ using the 2020 census-defined metropolitan and micropolitan statistical areas (Office of Management and Budget, 2020) (search string: ‘weight loss near = [city]’) and reviewed all business that appeared in the Google Maps-based results. Two authors reviewed all webpages associated with each business to identify those advertising semaglutide, tirzepatide, or liraglutide for weight loss. We supplemented this location-based search with regular (non-location-based) Google searches for ‘compounded semaglutide [city],’ again using census-defined metropolitan or micropolitan statistical areas. For convenience, we limited this supplementary search to semaglutide after observing that all businesses identified in the first search and advertising a specific GLP-1 product advertised at least semaglutide. Sponsored advertisement results and business without a physical address in Colorado (online-only) were excluded. We conducted these searches between March 21 and April 12, 2024.

We confirmed that businesses advertising semaglutide, tirzepatide, or liraglutide were selling compounded versions and not only the branded products. First, two authors manually reviewed all webpages associated with each business website and iteratively developed several criteria for identifying businesses advertising compounded products. These criteria included (1) explicitly disclosing that the products are compounded, (2) indicating partnership with a compounding pharmacy, (3) disclosing that products are created in a licensed pharmacy or laboratory, (4) advertising a sublingual product or a product that combines GLP-1 with another ingredient (e.g. vitamin B), or (5) claiming that the product is ‘generic’. We can be sure that products meeting either of these last two criteria are compounded because no such FDA-approved branded products exist, and generic versions will not be available anywhere in the world until at least 2026 (Silver, 2024). For businesses that could not be confirmed in this way, one author called to confirm whether available products are compounded.

Data collected from the identified business websites included physical addresses, business type, highest reported staff credential, advertised GLP-1 products, whether businesses referred to FDA approval when describing their products, and whether businesses referred to their products as ‘generic’. Two authors reviewed all webpages associated with each business website to identify and extract this information. Business type was determined using self-reported business categories in Google Maps or language from website business descriptions.

The Colorado Multiple Institutional Review Board determined this study represented nonhuman subjects research (COMIRB #24-1145).

## Results

**Figure 1.**
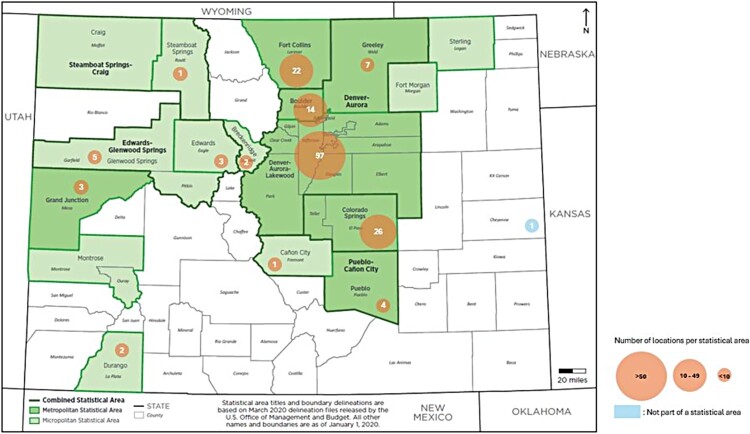
Physical locations of direct-to-consumer businesses that advertise compounded GLP-1 products for weight loss in Colorado. Note: Physical addresses are mapped according to the metropolitan or micropolitan area in which they are located (one location in Cheyenne County is not located in any metropolitan or micropolitan statistical area). Denver-Aurora-Lakewood: 97; Colorado Springs: 26; Fort Collins: 22; Boulder: 14; Greeley: 7; Glenwood Springs: 5; Pueblo: 4; Edwards: 3; Grand Junction: 3; Durango: 2; Breckenridge: 2; Canon City: 1; Steamboat Springs: 1; Cheyenne County: 1. Source of original map: https://www2.census.gov/programs-surveys/metro-micro/reference-maps/2020/state-maps/08_Colorado_2020.pdf

We identified 93 unique business websites advertising compounded GLP-1 products online and for sale in Colorado, a state with a population of approximately 5.8 million. Using information disclosed on their websites as described above, we confirmed that 38 of these businesses offer compounded GLP-1 products. For all but 5 of the remaining business, we confirmed by phone that their products are compounded. These 93 businesses correspond to 188 physical locations throughout the state (Figure 1).

Table 1 summarises characteristics of the businesses, available compounded GLP-1 products, and the language used to describe these products. Thirty-three (35.5%) businesses were self-categorized as medical or health spas, 26 (28.0%) were weight loss services, 11 (11.8%) were general wellness clinics, 7 (7.5%) were at-home mobile or telehealth services, 7 (7.5%) were individual physician or physician groups, 7 (7.5%) were anti-aging/regenerative medicine/hormone replacement therapy clinics, and 2 (2.2%) were men’s health clinics. Thirty-nine (41.9%) businesses stated that they included a medical or osteopathic doctor on staff, 10 (10.8%) included a nurse practitioner, 8 (8.6%) included a registered nurse, 32 (34.4%) did not indicate the type of training held by staff, and 4 (4.3%) included another type of credentialed individual (including a dietician, medical esthetician, chiropractor, and a health practitioner diplomate in the clinical science of anti-aging by the American Board of Anti-Aging Health Practitioners and the American Academy of Anti-Aging Medicine). Nearly all (98.9%) identified businesses advertised semaglutide products, 40 (43.0%) advertised tirzepatide, 2 (2.2%) advertised liraglutide, 1 (1.1%) advertised retatrutide, and 1 (1.1%) advertised an unspecified GLP-1 product. Eight (8.6%) businesses advertised products compounded with B vitamins and 1 (1.1%) each advertised products compounded with levocarnitine, mannitol, BPC 157, and glycine. Seven (7.5%) advertised oral formulations, including both tablets and sublingual troches. There were no businesses advertising salt forms of these products. Additionally, 41 (44.0%) of businesses referred to FDA approval in their descriptions of advertised products and 5 (5.4%) referred to their products as ‘generic’.

**Table 1.** Characteristics of business websites advertising compounded GLP-1 products and advertised products.

Characteristic	Businesses (*N* = 93)
***Business type***	**No. (%)**
Medical/health spa	33 (36)
Weight loss clinic	26 (28)
General wellness clinic	11 (12)
At-home (mobile/telehealth)	7 (8)
Individual physician/physician group^a^	7 (8)
Anti-aging/regenerative medicine/hormone replace therapy clinic	7 (8)
Men’s health clinic	2 (2)
***Highest level of provider credentials***	**No. (%)**
MD/DO	39 (42)
Nurse practitioner	10 (11)
Registered nurse	8 (9)
Other^b^	4 (4)
Unknown	32 (34)
***Advertised GLP-1 ingredient***	**No. (%)**
Semaglutide	92 (99)
Tirzepatide	40 (43)
Liraglutide	2 (2)
Retatrutide^c^	1 (1)
Unspecified GLP-1	1 (1)
***Ingredients combined with GLP-1 ingredient***	**No. (%)**
B vitamins	8 (9)
Levocarnitine	1 (1)
Mannitol	1 (1)
BPC-157^d^	1 (1)
Glycine	1 (1)
***Businesses advertising oral formulation***^c^	7 (8)
***Businesses referred to ‘FDA approval’***	41 (44)
***Businesses referring to product as ‘generic’***	5 (5)

Note: Percentages may not add to 100 due to rounding.

^a^ Includes 2 family practice physicians, 2 plastic surgeons, and 1 osteopath, OB-GYN, and primary care provider.

^b^ Includes a dietician, medical esthetician, chiropractor, and a health practitioner diplomate in the clinical science of anti-aging by the American Board of Anti-Aging Health Practitioners and the American Academy of Anti-Aging Medicine

^c^ Not commercially available for weight loss

^d^ Identified by the FDA as not safe for compounding

### Exploratory findings

We observed other notable claims on these websites that we record here as exploratory findings to inform future systematic research. Several websites made claims about the benefits and safety of these compounded products. For example, one website claimed several benefits of semaglutide that go beyond its FDA-approved indications including hair loss, stress, decreased immunity, and low muscle tone. Two websites claimed that combining semaglutide with a B vitamin can help reduce nausea side effects. Another asserted that combining its product with BPC-157 can mitigate many of the potential side effects of semaglutide. Another claimed: ‘this medicine works better when it is custom blended in a licensed laboratory.’ One provider of an oral formulation of compounded semaglutide claimed that ‘[t]roches have all the same risks and benefits as injectable semaglutide.’ The FAQ page of one website, in response to the question ‘Is it safe?’, stated, ‘Yes! The only studies showing adverse reactions were in lab rats.’

## Discussion

In this cross-sectional analysis, we found that compounded GLP-1 products for weight loss are available across Colorado. Most businesses advertising these products are self-classified as weight loss services or medical or health spas. The most commonly available product is compounded semaglutide, followed by tirzepatide. One provider advertised compounded retatrutide, a substance that is not FDA approved for any medical indication and is therefore not commercially available. While phase 3 trials for retatrutide are ongoing, these are not expected to be complete until 2026 (Lilly, 2024).

We also identified 7 businesses advertising oral formulations of compounded GLP-1 products. Currently, there is no oral GLP-1 drug approved for weight loss. Due to differences in the bioavailability of oral versus injectable formulations, further phase 3 trial evidence is needed to establish both efficacy and safety. Moreover, appropriate dosing should be established before oral compounded formulations should be advertised and sold.

While combination products (e.g. semaglutide compounded with B12) were not commonly advertised, we identified one provider selling semaglutide compounded with BPC-157. The FDA has stated that BPC-157 may pose an immunogenicity risk, and due to a lack of adequate safety-related information, would consider taking regulatory action against 503A compounders that compound drug products that include BPC-157 (U.S. Food and Drug Administration, 2024e). It is possible that combination products like these are more prevalent than can be determined from a website review alone.

We did not identify any businesses advertising salt forms of compounded GLP-1 products, and several websites explicitly stated that their products did not include these salt forms. Given that media reporting in May 2023 had identified the availability of such products, this finding can be seen as potential evidence of the effectiveness of letters sent by the FDA in late April and October 2023 to the National Association of Boards of Pharmacy and the Federation of State Medical Boards indicating that compounding these salt forms would not meet the requirements of the FD&C Act (U.S. Food and Drug Administration, 2024d). The FDA should consider further communication or action regarding other problematic practices in this market, such as the use of BPC-157. Further research is needed to establish the exact chemical nature of available compounded GLP-1 products.

Nearly half of the identified businesses referred on their websites to FDA approval when describing their products. This language is misleading because compounded drugs are not reviewed for safety and efficacy by the FDA and, unlike approved drugs, are not necessarily produced in facilities subject to Current Good Manufacturer Practice (CGMP) regulations. Only 503B compounders are routinely inspected by the FDA and subject to CGMP regulations; traditional 503A compounders are primarily under state board of pharmacy oversight. Future research should seek to identify from which types of compounding facilities businesses selling compounded GLP-1 products have sourced their product. This information would be useful for state boards of pharmacy wishing to inspect the manufacturing processes of traditional compounders who are supplying this product. We did observe a small number of websites that explicitly noted that compounded products are not FDA approved and that FDA approved branded alternatives exist. Although we did not systematically assess websites for such a statement, it is noteworthy that this type of disclosure was not prominently displayed across identified websites. Five websites also misleadingly referred to their compounded products as ‘generic’. Generic drugs are approved by the FDA after demonstrating bioequivalence to a branded product (U.S. Food and Drug Administration, 2024f); compounded drugs are not subject to this requirement. It is also important to note that many websites did not explicitly disclose that their GLP-1 products are compounded. Potential patients should be informed about the differences between branded and compounded versions of these drugs.

While we did not comprehensively analyze websites to identify claims about the safety and benefit of compounded GLP-1 products, an exploratory analysis did uncover examples of such claims that could be considered false or misleading. For example, it is false to claim that the only studies showing adverse reactions of semaglutide were in lab rats. It is also false to claim that semaglutide can be used to treat hair loss; rather, hair loss is noted in the FDA prescribing information as an adverse reaction of using semaglutide for chronic weight management (U.S. Food and Drug Administration, n.d.). Future research should establish the prevalence of false or misleading claims regarding safety and benefits on these websites.

In general, false and misleading advertising about consumer products is prohibited in the US and overseen by the Federal Trade Commission (FTC). However, the FDA – and not the FTC – has primary jurisdiction over the regulation of prescription drug advertising (Feldman, 2022). Moreover, FDA advertising regulations apply only to ‘manufacturers, packers, or distributors’, designations that do not appear to capture the novel business entities, such as telehealth platforms or medical spas, that are now commonly advertising and prescribing prescription drugs to consumers (Moore & Alexander, 2023). Policymakers should consider updating these regulations to apply more broadly to any business entity that prescribes or sells prescription drugs so that potential consumers of compounded medications are not supplied with false or misleading statements regarding their safety or efficacy, such as those noted above. Additionally, US lawmakers and regulators should consider amending the Federal Food, Drug, and Cosmetic Act or pursuing a Memorandum of Understanding to permit a coordinated regulatory effort by the FDA and FTC to oversee prescription drug advertising (Feldman, 2022). A coordinated effort would increase the resources currently available to the FDA to regulate the rapidly growing world of prescription drug advertising.

Given that more than one-third of the businesses identified in this study are self-classified as health or medical spas, accurate identification and oversight of medical spas represents another option for improved regulation of businesses selling compounded GLP-1 products. Medical spas typically offer non-surgical cosmetic procedures – such as botulinum toxin injections, laser hair removal, and body contouring – and are widely under the regulatory purview of state agencies (Yocale, 2023). In Colorado, medical spa practitioners must be licensed physicians or advanced practice registered nurses with prescriptive authority. Moreover, medical spas in Colorado may not make false or misleading claims about the benefits of their services (Yocale, 2023). Almost half of the businesses identified did not clearly identify whether a licensed prescriber (i.e. medical or osteopathic doctor, physician assistant, or nurse practitioner) was on staff. Therefore, it is unclear whether essential safety assessments are being provided such as identifying contraindications and drug interaction screening.

This study has several limitations. First, we relied on Google searches to identify the study sample. This approach may not have captured all businesses advertising GLP-1 drugs for weight loss. However, we chose a location-based search engine approach to replicate the way in which many potential customers across the state are likely to identify these businesses. Second, the market for compounded GLP-1 products is likely evolving; as such, the cross-sectional findings presented here are not generalisable across time. Nonetheless, these findings represent a baseline against which future research can be compared. Third, this study was limited to information gathered from business websites. It is unknown what information these businesses directly provide to potential customers or what type of monitoring and oversight is provided for those who use these products. Finally, for a small number of businesses we were unable to confirm that they offer compounded products. However, given that none of the contacted businesses disconfirmed that they sell these products, we have confidence that the identification strategy was accurate.

## Conclusion

GLP-1 drugs for chronic weight management have been described as ‘game changers’ (Kolata, 2021). However, many people who could benefit from these drugs may struggle to access them due to high list prices, inadequate insurance coverage, and ongoing supply shortages. In response, a robust market for compounded GLP-1 drugs has developed in Colorado and likely nationally. While compounding is a well-established pharmacy practice that can mitigate the impacts of drug shortages or fill a need for patients with allergies or other reasons that prevent them from using an FDA-approved drug, it is important to ensure that compounded drugs are safely manufactured and used. This cross-sectional analysis identified several instances of unapproved drugs or formulations being compounded and advertised in Colorado. Additionally, one product was advertised as compounded with BPC-157, a substance that the FDA has determined to be unsafe for compounding (U.S. Food and Drug Administration, 2024e). Finally, this study identified numerous examples of false or misleading claims regarding the regulatory status, benefits, and safety of compounded GLP-1 products. Increased oversight by the FDA, FTC, or state agencies can help to ensure that the benefits of compounded GLP-1 products for weight loss outweigh the risk.
